# Retraction: Associations of MMP-2 and MMP-9 gene polymorphism with ulinastatin efficacy in patients with severe acute pancreatitis

**DOI:** 10.1042/BSR20160612_RET

**Published:** 2026-03-26

**Authors:** Zhen-Fu Li, Guo-Dong Zhen, Lian-Bin Zhao, Shan-Shan Wu, Ming-Yu Chen, Zhen-He Li, Sheng-Zhi Zhou

**Affiliations:** Department of Emergency, Yishui Central Hospital of Linyi, Linyi 276400, Shandong Province, P.R. China

**Keywords:** Efficacy, MMP-2, MMP-9, Polymorphism, Severe acute pancreatitis, Ulinastatin

This article is being retracted from *Bioscience Reports* at the request of the Editor-in-Chief and the Editorial Board. This follows the receipt of a notification from a reader, alerting the Editorial Board to irregularities in the Western Blots of Figures 1B and C.

Specifically, there are background details in Figure 1B that unexpectedly repeat, and a region of the CC column of Figure 1C has a smudged appearance. The Editorial Board also has concerns surrounding the authorship of the manuscript.

The authors have been contacted with regards to the retraction, but they have not responded to the Journal's queries or the concerns raised. Given the extent of the issues raised, the Editorial Board stands by the decision to retract the article.

